# Academic literacy development and professional identity construction in non-native English-speaking novice English language teachers

**DOI:** 10.3389/fpsyg.2023.1190312

**Published:** 2023-08-11

**Authors:** Mo Chen, Wenli Zhang, Qun Zheng

**Affiliations:** Department of Foreign Languages, University of Chinese Academy of Sciences, Beijing, China

**Keywords:** academic literacy, professional identity, Community of Practice, novice teacher, non-native English-speaking

## Abstract

Studies examining students’ academic literacy development have received increasing attention in the past two decades, with exponential growth in the literature since 2010. Despite this, there have been relatively few empirical studies on novice teachers’ academic literacy learning process and the construction of teachers’ professional identities. To address this issue, this study employed a longitudinal narrative inquiry to trace and evaluate the professional identity construction of two Chinese novice language teachers as they developed academic literacy in their master’s and doctoral programs in the United States. The study adopted a Community of Practice (CoP) framework and drew upon various data sources, such as both novice teachers’ coursework, reflection journals, statements of teaching philosophy, and other supplementary documents, to evaluate how each participant was socially engaged (or disengaged) in a new language education community and how they developed professional identities as language teachers in this process. The findings indicate that the pedagogical knowledge and theories acquired by both participants during their graduate studies informed their teaching methods and promoted their development of a researcher-teacher identity. In addition, the longitudinal data allowed for tracking changes in both participants’ self-efficacy and emotions. While the stories of both novice teachers described the evolution of their teaching beliefs throughout their graduate studies, their narratives also highlighted a lack of micropolitical literacy training at the graduate level. This study contributes to our understanding of the connection between academic literacy development and the formation of teacher professional identity by shedding light on novice language teachers with a non-native English-speaking background. The implications for future research are also provided.

## 1. Introduction

Academic literacy, considered a powerful social practice for knowledge acquisition, skill generation, and self-transformation, has been receiving increased attention in the language education domain. Traditionally, academic literacy refers to “the ability to read, write, and compute in the form taught and expected in formal education” (Ogbu, 1990, p. 37). Many early studies grounded in behaviorism mainly focused on academic literacy instruction and remedial classes, evaluating the effects of teaching essay reading and writing, note-taking, and general language skills on non-native English-speaking (NNES) students’ language acquisition. Currently, the enhanced understanding of academic literacy associated with constructivism has framed this concept as a multifaceted and complex construct. Recent research has posed discipline-based and sociocultural approaches to explore the construct at the levels of disciplinary epistemology, power struggle, and identity construction (Gee, 2014).

As the concept of academic literacy has expanded, its interconnectedness with the notion of identity has received increased attention (Lea and Street, 2006). Some studies have explored how students’ identity formation relates to their academic literacy development and how their learning experiences and socio-academic relationships in a new discourse community affect the construction of their identities (e.g., Bilikozen, 2015). By comparison, empirical studies on teachers’ professional identity (PI) construction, along with their academic literacy learning processes, are less abundant. Given that many studies have demonstrated that teachers’ robust and stable PI is linked to quality instruction and improved emotional well-being of teachers and students (e.g., Agee, 2004; Hong et al., 2017), it is meaningful to explore the extent to which academic literacy development relates to the construction of teachers’ PI.

This study adopts a Community of Practice (CoP) framework (Wenger, 1998) to analyze the identity construction of two Chinese novice language teachers when they were acquiring academic literacy in master’s and doctoral programs in American universities. Data collection spanned over 8 years and included course and practicum journals, reflective journals, emails, and personal statements to evaluate how each participant was socially engaged or disengaged in their CoPs and how they evolved PIs in this process. The findings have illustrated the trajectories of the participants’ PI construction in overseas institutions and highlighted the important factors and experiences that contributed to novice teacher perspectives on academic literacy acquisition. This study has also added to our understanding of the relationship between academic literacy development and the formation of teachers’ PI, shedding light on the experiences of novice language teachers with NNES backgrounds.

## 2. Literature review

### 2.1. Academic literacy models and research on the academic literacy development

Academic literacy, which encompasses various forms of literacy in academia, is a complex concept (Street, 2003). Lea and Street (1998) proposed three models to explore students’ academic literacy development: the study skills model, the academic socialization model, and the academic literacies model. The study skills model, derived from traditional definitions of academic literacy, focuses on decontextualized, transferable reading and writing skills. It is concerned with instructing students on formal language features such as sentence structure and grammar. The second model, academic socialization, emphasizes situated learning and familiarity with the norms and communication styles specific to a particular discipline. The third model, academic literacies, takes a more comprehensive approach by evaluating meaning-making in social practice and examining power, identity, and authority at the institutional level.

The pioneering efforts to address academic literacy with these models have inspired considerable exploration of aspects of literacy development. Some researchers have investigated academic literacy development from the aspects of language use and language competence development in academic settings (e.g., Baumann and Graves, 2010; Sebolai, 2016). Other authors have discussed discipline-specific language, along with cognitive and social practice (e.g., Lillis and Scott, 2007). Additionally, studies have addressed academic literacy from a broader perspective, exploring power and social justice (e.g., Leki, 2007; Gee, 2014). Those studies have generated a deeper understanding of the intricate interaction between multiple sociocultural factors that contribute to academic literacy development.

While current research on academic literacy is substantial and informative, one central challenge concerns the time frame. As Lillis and Scott (2007) pointed out, academic literacy development is fluid and changes over time; however, most studies were conducted within a relatively short period ranging from 1 month to 1 year, failing to capture literacy progression in the long term. In comparison, longitudinal studies recording participants’ dynamic learning experiences, sociocultural practice, and evolving ideological views remain uncharted. Spack’s (1997) study is one of the few endeavors unveiling how an NNES student’s acquisition of academic literacy shaped her perspective toward American social practices. Considering the importance and challenges of acquiring academic literacy for NNES students and the limited longitudinal research on this topic, we have found great value in exploring the long-term academic literacy development of NNES English learners.

Moreover, academic literacy research can be expanded to encompass under-explored areas and participants (Yeh, 2011). While there have been studies in various disciplines, such as engineering (Koutsantoni, 2006), mathematics (Mongillo, 2017), and business (Zhao and Chan, 2014), there has been limited attention given to the domain of language teacher education, such as Teaching English to Speakers of Other Languages (TESOL) programs and Applied Linguistics programs. In these programs, students not only learn theoretical knowledge of language learning and pedagogical skills and strategies, but also work as teachers in language classrooms under the supervision of their mentors or supervisors. We found it worth investigating how novice teachers in these programs develop various forms of academic literacy that prepare them to be qualified language teachers and impart literacy knowledge and skills to their students.

### 2.2. The research on teacher’s PI

One concept related to literacy development is identity formation. According to Egbo (2004), identity refers to the ways individuals view themselves and their societal status, especially when they experience unequal treatment and social injustice. Teachers’ PI, therefore, is not simply about self-perception as teachers, but also entails an integration of self-experience, collective practice, and personal belief (Zhang and Wang, 2022). Scholars have found that a strong PI can enhance teachers’ confidence and commitment to their profession and decision-making, ultimately improving the quality of classroom teaching (Beauchamp and Thomas, 2009). Reviews on PI have revealed a growing research interest in the development of novice teacher PI in the past decades. According to Lortie (1975), novice teachers in graduate education programs are in a crucial developmental career stage in which they learn theoretical and pedagogical knowledge in graduate programs, apply them in practicum settings, and gradually form their self-image and identity. Analyzing the views of novice teachers about PI can uncover any fears or misguided expectations and offer a guide for building the abilities or tendencies necessary to achieve their desired teaching role. Some studies have documented the cognitive, emotional, and social processes of developing and modifying PI in teacher education programs and recorded the reciprocal way that personal and social factors interact to shape PI (e.g., Lasky, 2005; Avraamidou, 2014). However, these studies mainly focus on pre-service teachers trained for secondary education. The PI development of novice teachers for higher education, however, has been underexplored.

Studies examining teachers’ PI in different disciplinary fields have also revealed that the topic of NNES language-teacher identity has received increasing attention in the past two decades, with exponential growth in the literature since 2010. This growth can be attributed to the increasing number of NNES teachers pursuing their graduate degrees in TESOL, Applied Linguistics, and other teacher preparation programs. Researchers found these novice teachers not only struggle with their dichotomized and transformative roles as both language learners and professionals due to their accents and language competence in education contexts, but they may also experience racial mistrust and inequity due to power disparity and cultural stereotypes both inside and outside the classroom (Moussu and Llurda, 2008). As a result, a body of research has investigated the challenges these novice NNES teachers face in their early learning stages when developing a legitimate teaching status. For example, Moussu and Llurda (2008) discovered that people’s perceptions of linguistic competence have led them to categorize language teachers as native or non-native speakers, which can pose problems in teacher education. This dichotomy can lead NNES teachers to feel inferior and question their legitimacy as TESOL professionals. Some researchers have taken a sociopolitical perspective to evaluate the formation of language teachers’ PIs. They have looked into the interaction between individual and social factors and found that NNES teachers’ PIs can be undermined by some inherently racialized TESOL programs and structured inequity in academia and society (Hayes, 2009).

It is noted that many of these studies have explored language teachers’ identity development during their practicum (e.g., Zhu and Chen, 2022) or within a one-semester, short-term training course (e.g., Soong et al., 2021). While the data collected by these researchers have captured invaluable identity construction experiences and insights from participants, they have been unable to outline the trajectories of teacher identity development in language teaching programs and the factors that influence it. Tsui (2007) and Liu and Xu (2011) are two studies that illustrate the shift of language teachers’ identity through narrative inquiry in English-as-a-foreign-language contexts. However, the longitudinal research focusing on English language teacher identity in English-as-a-second-language (ESL) contexts is minimal. According to Llurda (2005), 36% of students in TESOL graduate programs in North America were NNESs. The ways in which these students perceive themselves as professionals can affect their communication with their colleagues, their institutions, and their own students, as well as their pedagogical implementation and classroom management, thus affecting their career development.

According to Lea and Street (2006), academic literacy, which involves knowledge learning, meaning making, and power and authority understanding, can influence identity construction in academia. Based on this belief, some researchers have explored NNES English language learners’ challenges in forming their new identity in a new discourse community when acquiring academic literacy (Choi, 2019; Donovan and Erskine-Shaw, 2020). However, the research on the literacy development of novice language teachers with NNES backgrounds in graduate teacher preparation programs and their PI formation is lacking. As Li (2007) mentioned, NNES English language teachers always encounter identity confusion and cultural clashes when they are aware of their identities as both language learners and teachers. Considering the challenges NNES English language teachers encounter and the minimal research on this group of people, we found it meaningful to conduct this study, collecting qualitative data to investigate two NNES novice language teachers concerning their academic literacy development in relation to their identity construction. This study provides novel insights into the academic literacy development of novice teachers in the field of language education.

### 2.3. Theoretical framework

This study employs Communities of Practice (CoP), a sociocultural theory of learning (Wenger, 1998), to investigate both novice teachers’ PI construction. CoPs are defined as “groups of people who share a concern or a passion for something they do and learn how to do it better as they interact regularly” (Wenger-Trayner and Wenger-Trayner, 2015, p. 2). Novice teachers become active participants in their CoP through cooperation with other members (i.e., their peers and advisors); moreover, their development is socially situated in their CoP since learning to teach, which involves teachers learning academic literacy, is a socially mediated activity (Lave and Wenger, 1991; Feryok, 2012; Johnson, 2016).

According to the notion of situated learning in CoP, learning is a process of identification, which elucidates the connection between learning and identity construction. The relationship between learning and identity is characterized as mutually constituting: “learning is, in this purview, more basically a process of coming to be—of forging identities in activities in the world” (Lave, 1992, p. 3). In other words, identity is socially constructed through learners’ active participation in the practices of social communities (Wenger, 1998). In view of this, teacher PI is a critical component of teacher learning and academic literacy development and is socially formed in situated contexts (Lave and Wenger, 1991; Varghese et al., 2005). In other words, identity formation is contextualized in teachers’ CoPs.

CoP has been implemented in many domains, including examining academic literacy and identity development. Researchers (e.g., Hirvela and Yi, 2008; Cho, 2013; Vallente, 2020) have employed CoP to analyze students’ academic socialization in their academic contexts, including their identity development. Nevertheless, very scarce literature has holistically and comprehensively explored the PI development of novice teachers.

Teacher PI is formed through pedagogical training and constructed in social settings (Varghese et al., 2005; Vallente, 2020), and the sociocultural theory of CoP allows researchers to explore the construction and developmental process of teacher professional identity in formal educational programs. In this sense, CoP offers a theoretical lens for researchers to understand teacher learning as the process of becoming a teacher. In this study, graduate programs served as professional CoPs in which both the researchers learned to teach and grew into professional language teachers. The use of CoP allows for a profound analysis of how both novice teachers’ learning in graduate programs and their academic literacy development influenced their professional identity construction. In this study, we aimed to address one research question:

How was NNES novice language teachers’ academic literacy development related to the formation of their PIs in the master’s and doctoral programs?

## 3. Methods

This study employs narrative inquiry as the methodology. Researchers found that analyzing people’s narratives can provide in-depth insights into their experiences (Bruner, 1987; Connelly and Clandinin, 1990, 2005; Atkinson, 1997). Therefore, narrative inquiry has been highly acknowledged and extensively adopted in many academic fields. In education, teachers are also storytellers of their experiences. Narrative inquiry can examine teacher development, as it allows researchers to comprehensively describe participants’ experiences and create complete portraits of them (Connelly and Clandinin, 1990; Bell, 2002; Johnson and Golombek, 2011). By adopting narrative inquiry in this study, we can investigate how both participants’ PIs have evolved over time.

### 3.1. Participants and contexts

The study participants were Irene and Rose (the first two authors of the study), both Chinese female lecturers at a research-oriented university in China who go by their English names. Irene earned her bachelor’s degree in English language and literature in China before moving to the United States where she completed two master’s degrees: one in TESOL and one in Applied Second Language Studies. While in the TESOL program, Irene served as a teaching assistant for a year and taught English academic writing and beginner-level Mandarin Chinese. In 2012, she joined a doctoral program in Applied Linguistics and taught academic writing courses at intermediate and advanced levels for 5 years. Rose obtained her bachelor’s degree in English in China and worked as an English teacher in a cram school for nearly 6 months before moving to the United States for her graduate studies. After finishing her master’s degree in the Teaching English as a Foreign Language program, Rose taught an English academic writing course for first-year international students for one academic year. In 2014, she began her doctoral-level study at a large state university and received training in language education. While pursuing her doctoral degree, Rose worked as a writing consultant in the university’s writing center for 3 years, serving native and international students from different backgrounds.

Irene and Rose are both the researchers and participants in this study. We recognize that taking on both roles can be a limiting factor as it may not provide sufficient detachment for objective analysis. However, as Radnor (2001) pointed out, assuming dual roles can also be a positive aspect when researchers are actively engaged in interpretive research and aware of their potential bias that could influence the study. We also believe that the experiences as NNES students and novice language teachers in TESOL and Applied Linguistics master’s and doctoral programs in the United States for more than 8 years are invaluable. We represent the teaching and learning experiences of a group of NNES students and novice teachers in graduate programs, enabling us to trace academic literacy development over the years as insider researchers.

### 3.2. Materials

The data for this paper were derived from two sources. The first source consisted of 37 reflective journals, with 30 of them documenting the reflections and thoughts of Irene and Rose during their graduate programs’ coursework and teaching practicum for over 8 years. Seven of these journals were written after they completed their doctoral degrees, reflecting on their overall learning and teaching experiences. The second data source is supplementary documents, including 26 course assignments, three training notes, two teaching philosophies, one cover letter for job applications, and one email. The course assignments, teaching philosophies, and cover letter mainly outlined the beliefs, values, and principles guiding Irene’s and Rose’s teaching practice. The training notes recorded thoughts, questions, and ideas about teaching before and after teacher training. The email was included as it captured one participant’s communications with professors regarding her academic development and personal queries about graduate programs.

### 3.3. Research procedure

During the first 5 weeks of the study, Irene and Rose gathered their own reflective journals and related documents that recorded their experiences in the master’s and doctoral programs. They both saved these files in a folder in chronological order and wrote additional journals to reflect on their prior experiences in the graduate programs. Then, Irene and Rose wrote their stories based on their journals and other documents, recording their academic literacy development in the graduate programs and reflecting on the formation of their own PI as teachers.

### 3.4. Data analysis

The research team conducted a narrative analysis of the collected data to answer the research question concerning NNES novice language teachers’ academic literacy development and PI formation throughout their graduate school journey. This included a holistic examination of course assignments, self-reflection journals, and other sources and the use of notes to identify descriptions related to academic literacy and teacher identity. The research team then engaged in member checking to verify interpretations and refine notes to identify broad themes in the analysis (e.g., self-perception as teachers, micropolitics, beliefs about teaching, emotion, and self-efficacy). Given Irene’s and Rose’s insider status as both researchers and participants, the member checking process allowed them to gain outside perspectives, assess biases, and triangulate findings. Following analysis and member checks, Irene and Rose assembled their narratives, structuring them with exposition, complication, climax, and resolution, as recommended by Gordon and Kuehner (1999). The exposition of a story entailed delivering the background of each narrative. The complication described the participants’ practices as novice teachers in classrooms, and the climax section depicted the participants’ confusion and difficulties as NNES novice English language teachers in ESL contexts. The resolution section outlined the participants’ solutions to and thoughts on the problems and their previous concerns. To illustrate their academic literacy development and PI formation, Irene and Rose used notes to triangulate multiple data sources into their mini-stories within the broader narratives. Once complete, the team reviewed the triangulated narratives to strengthen credibility and ensure a balanced portrayal beyond selective narration.

## 4. Results

### 4.1. Irene’s story

In this section, Irene analyzes her narratives to illuminate her path to becoming a language teacher. The overall trajectory depicts the development of her academic literacy and the growth of her PI during her graduate studies. The following story was written by Irene using first-person narration.

#### 4.1.1. Literacy and communication challenges in the teacher education program

I received my bachelor’s degree in English Language and Literature in China. The three-month teaching practicum in the fourth year of my undergraduate program sparked my interest in becoming a language teacher. In 2008, I enrolled in my first master’s program at a public university in the United States, majoring in TESOL within the teacher education department. Although I was excited and enthusiastic to introduce myself to my classmates during the first week, I soon found the course reading assignments and communication overshadowed my joy of joining the program. Many of the courses required extensive reading of textbook chapters and research articles for weekly class discussions. I found the reading materials to be “very long, dense, obscure, and full of theoretical terms in education and psychology” (Reflection Journal #3). Despite my efforts to complete the reading assignments before class, I realized that I was not an efficient or strategic reader. As documented in my personal journal:

[Before class,] I spent hours upon hours reading book chapters and journal papers, but I still struggled to comprehend the essence of the authors’ messages. Then I found that I was focusing too much on new words and finer points of articles that the professor did not emphasize. (Reflection Journal #4).

Furthermore, I came to realize that my lack of critical thinking skills was impeding my ability to question ideas presented in texts and adequately prepare for classroom discussions. My classmates were proficient at challenging the ideas presented in the texts and contributing their own learning and teaching experiences to further the discourse. However, I found myself unable to do so. One reason for this was my tendency to focus solely on comprehending the authors’ viewpoints, without engaging in critical thinking. Additionally, due to my introverted nature, I required time to formulate my thoughts before participating in class discussions.

Apart from the challenges with reading and critical thinking, my lack of familiarity with teaching and classroom communication in the teacher education program also hindered me from joining the new discourse community. In the program, the instructors not only imparted knowledge through traditional lectures but also employed a variety of class activities, such as group discussions and presentations, to foster student engagement and learning. During one of these sessions, a professor introduced different educational philosophies. After his lecture on the subject matter and related theoretical orientations, we formed groups of three or four, and each group was tasked with exploring and presenting one theory. Many of my classmates were experienced teachers who were able to draw from their practical knowledge to illustrate theories in an easily comprehensible manner. As a novice with limited theoretical knowledge and teaching experience, I was apprehensive about delivering an impromptu speech to the class: “I believe that my classmates enjoyed the class, as they often shared personal stories and shared laughter, but I often felt like an outsider, sitting quietly in the corner of the classroom and waiting for the end of class.”

A sense of unease and a lack of participation induced considerable anxiety on my side and rendered me unable to integrate into the teacher community. It was not until the second semester of the program when I took a course in sociolinguistics and met the professor, who had migrated from China to Canada in her early 20s, that my situation improved. The professor illustrated various difficulties she had faced while adapting to the environment as an NNES student in the teacher education program. She also encouraged me to interact with her and my classmates by sharing stories with them. When I took my first major communicative leap—commenting on my classmates’ ideas and sharing with them my English learning experience at home and school—I soon realized that my classmates were curious about the linguistic and sociocultural problems I had encountered as an NNES, as well as the educational environment and policies in China. This new sense of value as a contributor to the learning community helped me overcome my initial unease and become more engaged in the class. I also met the professor in her office to discuss my research interests and ask questions about assignments in more detail. Engaging with classmates and faculty helped me feel more connected to the community and provided valuable support and resources throughout my studies.

#### 4.1.2. Teacher belief development and teaching dilemmas in the practicum

I undertook my teaching practicum during the third semester of my TESOL program. This course provided practical experiences for students to apply the knowledge they had gained in graduate-level courses. During my practicum, I conducted 10 weeks of class observations and taught one solo class in an ESL speaking course. As part of the course assignments, I wrote five reflective journals. These practices and self-reflections allowed me to gain valuable insights into the practical application of various teaching techniques and approaches in real ESL classrooms. As I mentioned in one journal,

In this week’s speaking class, students first worked in groups to discuss different academic etiquettes in the U.S. and then gave a 5-min presentation using the knowledge learned in the class. When a student ended their presentation, the teacher always commented on the strengths of the speech and then provided constructive feedback. I think this is how constructivist learning theories were applied in the classroom. Students learned in a cozy and secure community, and the teacher facilitated their learning and showed empathy and support with the goal of helping the person improve rather than making them feel embarrassed in class. (Course reflection #2).

My teaching belief was continually strengthened throughout my teaching practicum in my second master’s program of Applied Second Language Acquisition with a focus on Chinese language pedagogy. One main feature of the teaching practicum in this program was to provide novice teachers with extensive opportunities to observe various language courses at different proficiency levels. In one advanced Chinese language and culture course, entitled “Ghost Stories from Chinese Literature,” students read supernatural and imaginative stories with East Asian philosophies and romance in the Chinese classic “The Strange Tales from Liaozhai.” In the class, they identified and compared the cultural values and beliefs infused in those stories and compared them with those from their culture. They were also involved in creative writing, utilizing the language and knowledge they learned to continue the stories in Liaozhai. Communication with the professor allowed me to understand that his teaching philosophy centered around the ideas of cultural competence and communicative competence.

This course blew my mind. Students learn about Chinese culture and society through the lens of the Chinese language. I believe that language courses should help students develop their language skills in a meaningful and communicative learning environment. Language teachers, as cultural communicators, can help students become well-rounded and culturally aware individuals and prepare them for future academic endeavors and cross-cultural communication. (Course reflection #9).

As part of my teaching practicum, I also performed teaching demos and received feedback from my mentor and colleagues. Interestingly, my reflective journals not only captured my enjoyable teaching moments but also recorded my struggles when I had conflicting ideas with my mentors. Once, I was required to conduct a 20-min teaching demo in a beginner-level Chinese class. I first taught students basic vocabulary for family greetings and gatherings and then designed activities for practice and social norms acquisition. One of the main arguments between my mentor and me was about language use. I mainly spoke Mandarin Chinese to introduce new vocabulary and sentence structures but switched to English to explain Chinese customs, traditions, and values, and to provide feedback to students at the beginner level. However, my mentor believed that using English in the instruction had impeded students from immersing themselves in the Chinese language. At that time, I found it extremely difficult to advocate for myself or challenge my mentor’s feedback. In the reflective journal, I said,

My mentor was an experienced teacher whom I respected and learned a lot from, but we had a disagreement regarding language use in teaching Chinese. She believed that using Chinese instructions when teaching Chinese would benefit students the most, while I felt that the decision to use English or Chinese instructions should be made on a case-by-case basis, taking into account the student’s proficiency level and the teacher’s teaching approach and philosophies. Although I tried to clarify my thinking, I was also worried about the power dynamic with my mentor and the impact of my word choices. (Self-reflection #2).

Reflecting on this experience, I realized that challenging one’s mentor can be a valuable part of the learning process and can lead to growth and development in teaching practice. However, the teaching practicum is a high-stakes environment, and novice teachers are under pressure to demonstrate their competence and meet performance standards, which can exacerbate the challenges of navigating power relations with a mentor. Therefore, it was important to acquire the necessary academic literacy to find areas of agreement, share my perspectives, and communicate respectfully and professionally with the mentor. The practicum allowed me to be aware of the micropolitical reality and my lack of competence and skills to communicate with mentors, administrators, or others who had more power.

#### 4.1.3. Emotional growth and teacher identity formation as a novice teacher

Equipped with theoretical and practical knowledge gained through various courses, I began my teaching assistant journey in 2010 during my first master’s program. While I had training in my practicum, I experienced emotional burnout throughout the semester as I taught independently for the first time. I always took a deep breath before entering the classroom, and when I spoke the first word in front of my students, I felt extremely nervous with sweaty hands and a trembling voice. Moreover, I was always worried about technology failure.

I have heard stories about technology failures during teaching. Teachers who have spent hours crafting good lessons with the integration of videos and technology-infused peer review often find themselves disappointed when technology fails. For instance, the instructor’s computer may have no sound, the internet connection may go out, or the apps used for class activities may not work. I would feel terrible if that happened in my classroom. (Self-reflection # 1).

As evidenced in my reflection, I also found it difficult to cater to diverse learning needs.

Some students were more capable of completing tasks given by the instructor, while others needed more support and explanation. When I am the only instructor in the classroom, I find it difficult to differentiate instruction to meet all their needs. (Self-reflection # 1).

Being a member of the teaching community has offered me a sense of belonging and support, which was especially valuable to me as a novice teacher experiencing stress and anxiety. During bi-weekly instructor meetings, experienced colleagues shared various activities they integrated into the classroom and instructional strategies they used to manage the class. When I shared with them that my students often said I looked too young to be a teacher, many of my colleagues offered advice. One colleague, Sarah, suggested that I should dress more professionally to establish myself as a teacher, especially during the first month of each semester. Taking her suggestion, I found that professional clothes not only helped build appropriate relations with students but also adjusted my identity as a professional.

By dressing up for teaching, I saw myself more like a teacher than a student. I feel stronger and more authoritative in front of my students. The dressing reminds me of my identity and helps me keep my speech and body language professional. (Training note # 2).

In addition, learning from experienced colleagues, I prepared paper-based exercises in case of technology failure in the classroom. I designed peer review activities to encourage students’ interactive learning while being able to address questions and offer scaffolding to the students in need. The accumulated teaching experience, along with communication with my colleagues, allowed me to manage my stress and anxiety and enhance my belief in myself as a supportive teacher in the classroom.

My teaching journey and teacher identity were further enriched in the doctoral program of Applied Linguistics and Technology. Due to my research interests in computer-assisted language learning, I explored various Automated Writing Evaluation (AWE) systems and corpus-based tools for writing. These tools allowed students to take the initiative for their learning and promoted collaboration and problem-solving skills. As a teaching assistant, I have seen how such tools can be used to foster student engagement in discussion and knowledge exchange, thus enhancing the learning experience.

While teaching, I witnessed my dual role as a teacher and researcher in the classroom. My interests in corpus linguistics and educational technology have enabled me to draw on research-based pedagogical practices and incorporate the latest research findings into my classroom. Noticing the common syntactic errors made by ESL learners, I conducted research with my colleagues and assistants, building up a learner corpus with student writing in academic writing courses and identifying typical error patterns. Based on the findings, we developed a corpus-informed feedback system that provides feedback to address typical syntactic errors in writing. The reciprocal relationship between my teaching and research informed my new role in the class.

It took us about 2 years to complete this project. When the system is used for error correction exercises in real classrooms and receives positive feedback from students, I feel fulfilled and empowered because I can incorporate technology and research findings into course design to facilitate student learning. Students’ negative feedback has also helped me understand language and its complexities and improve the system. It’s exciting to work as both a teacher and researcher in the classroom. (Self-reflection #3).

In addition to developing a dual identity in the classroom, I gradually recognized the value of being an NNES as an English instructor. I used to believe that my NNES identity had inherent drawbacks for my profession. However, my teaching and learning experience has taught me about my unique strengths in teaching English courses. In the doctoral program, I read extensively on various research topics and wrote essays, proposals, term papers for course projects, and research work. This experience developed my note-taking and summarizing strategies to cope with my reading and writing tasks. Being aware of the literacy challenges that ESL students face, I integrated those strategies and skills into teaching and drew on my own struggles and successes to help students overcome common obstacles. I also understood the feeling of studying in a new academic setting with cultural differences. Therefore, I created a supportive and inclusive classroom environment where students feel comfortable sharing their concerns and asking for help. As recorded in my journal,

I still remember a female student from Saudi Arabia who cried and talked to me at the end of Spring semester 2016. She said that the English reading and writing course she took with me had been the most memorable course since she came to the United States. She initially felt uncomfortable in the classroom due to language barriers and cultural differences. But when I approached her and listened to her concerns, she felt cared for and secure. In the class, she started asking me questions and sharing her thoughts. She also visited me during my office hours to ask for suggestions for her writing and future careers. At the end of the semester, she said that she would go back to her home country soon, but she had a growth mindset to view challenges and setbacks in her learning process. (Self-reflection #1).

The positive feedback and suggestions from my students have boosted my confidence as a language teacher. I still dress professionally at the beginning of each semester because it can set a positive tone for the classroom and maintain a formal and respectful environment. However, dressing is not the main source of my professionalism. In fact, my five-year teaching assistant experience promoted my inner growth and enhanced my resilience and confidence in the class. I can stay calm to cope with the demands of teaching and the challenges that arise in the classroom and maintain my professionalism. While I am still in my early career, I have become more resilient, bouncing back from difficult situations, reflecting on underlying causes, and seeking support and advice from my supervisors and colleagues when necessary.

### 4.2. Rose’s story

This section describes Rose’s experience in becoming a professional language teacher through her training and work in her master’s and doctoral programs. In both graduate programs, she developed her academic literacy in the field of language education, and her growth as a responsive, reflective, and theoretically-grounded language teacher was facilitated. Rose’s experience also reflected her dual identity as a researcher in language education and a language teacher.

#### 4.2.1. The emergence of Rose’s PI as a language teacher

I was raised and educated in China and came to the United States from a culture in which teaching is often teacher-centered. That was the teaching model I was most familiar with as a student and an apprentice (Lortie, 1975). To train novice language teachers, the master’s program I attended offered content and methods courses on language education. These courses provided me with systematic and formal training in language teaching and accelerated my transition from a language learner to a language teacher. Several courses in the program influenced my teacher PI development.

My first methods course, Teaching ESL, changed my beliefs about what constitutes good teaching. In this course, I learned pedagogical theories and concepts about language learning and teaching and had opportunities to put these theories into practice. For instance, I was engaged in a team-teaching project that consisted of four stages: lesson planning, rehearsal, video-recorded teaching, and debriefing. During the debriefing session, we watched the video of our teaching with the instructor and reflected on our teaching practice. Afterward, I completed a reflection journal to deliberate on the entire process of the team-teaching activity. The reflection activity reinforced my understanding of the theories I had learned and raised my awareness of how I applied them.

Through the training in this course, I began to view myself as a responsive teacher who consciously adjusts their teaching according to students’ responses. This is evident in my reflection journal on the team-teaching activity. Throughout the project, I considered students and their learning and worked to make the lesson accessible and comprehensible to them. In my reflection journal on the team-teaching project, I expressed concerns about engaging students and making the lesson appealing to them:

We felt the pressure as we were afraid our lesson would not be appealing and that [students] may lose their interest… We learned that we, as teachers, should teach to the students… Knowing students would make the class more effective, as knowing their needs allows teachers to prepare for the class more specifically… [Students’ feedback] helps teachers figure out what to teach next and informs them about how students understand what they were taught. (Reflection Journal).

This statement reveals my belief that student learning lies at the heart of any teaching activity. I strove to consider student learning effects while designing and giving instructions and made an effort to create student-centered learning environments. This ideology infused my teaching. The instructor of the methods course commented on my reflection journal, stating that it provided insights into “how [I] see [myself] as a teacher and how [I] think about the activity of teaching” (written communication). Her comments demonstrate the emergence of my language teacher PI.

Reflecting on my teaching experience verbally and in written form helped me engage with intentional teaching and encouraged me to become a reflective and considerate teacher. As for the team-teaching project, the debriefing session and my written reflection created a space for me to better understand why I did what I did in teaching and how I might think differently about what I had done. Being reflective and introspective in teaching also promoted the formation of my dual identity as both a researcher and a practitioner. I expressed my appreciation of the opportunity to apply theories to practice in my reflections:

The team-teaching project was a really good chance for us to better understand teaching ESL students and applying the theories we learned. There are many differences between keeping theories in mind and applying them to practice. Initially, we might not know when to use a specific theory. We might misuse theories or not realize we were applying some theories to teaching. This project provided a very practical chance for us to experience how to apply theories appropriately. (Reflection Journal).

The debriefing session allowed us to review our teaching performance. The instructor helped us analyze the theories we applied to teaching, some of which we had not noticed. Furthermore, she gave us practical suggestions that could be used to improve our future teaching. (Self-Reflection, December 20, 2022).

These excerpts demonstrate my awareness of the importance of marrying theories with practice. In the reflection journal, I pointed out the value of the team-teaching project and its reflection activity which allowed me to connect theories and practice. The comments from the instructor suggest that my reflections show I understand what is necessary to be a good ESL/EFL teacher, demonstrating the development of my language teacher PI.

My PI development as a language teacher was improved through my practicum experience. In my final semester in the master’s program, I completed a semester-long practicum where I worked with an ESL instructor as a teaching assistant. I observed the instructor’s teaching, led activities, and taught lessons during the semester. The practicum supervisor observed and recorded three of my lessons, and a debriefing session was conducted after each observation. During the practicum, I learned from my observations of the instructor’s teaching and constantly communicated with her and the practicum supervisor, who were members of my CoP. They offered suggestions about lesson planning, content development, pedagogies, and language teaching in general. I wrote eight journals to reflect on my practicum experience. In one journal, I retrospectively analyzed an emotional moment in a lesson I taught at the beginning of the semester. I described the situation I had encountered and my feelings about it, and I also considered what could have been done to make this moment teachable. Through reflection, my thoughts on teaching were elicited, promoting my PI development.

My PI development was also supported by observing the ESL instructor’s teaching during the practicum. By employing methods such as questioning, grouping, and discussion, the instructor created a student-centered classroom that served as means for “comprehension checking, evoking group or pair work, eliciting students’ deep thinking, and encouraging students to talk more” (as noted in Reflection Journal #2), resulting in a significant increase in student participation and learning. I adopted these strategies into my teaching by integrating more questions and discussions to maximize the learning opportunities for students. This experience positively reinforced my development into a responsive language teacher.

My development as a reflective teacher was also furthered through the teaching activities during the practicum. For instance, while teaching argumentative essay writing, I noticed students’ confusion in understanding the concept of a counter-argument, so I adjusted my instruction and retaught this part to account for their reactions. As I reflected, “it was necessary for me to go back and spend more time explaining counter-arguments and refutations” (Reflection Journal #7) to promote students’ comprehension. I continued reflecting on the readjustment:

Sometimes, it is okay, even necessary, to shift pedagogical goals based on students’ learning effects and responses. A lesson plan, which works as a guide, can be really flexible… If sticking to a lesson plan rigidly, a teacher might easily ignore students’ reactions or learning during teaching, especially when students might have trouble understanding… If shifting the pedagogical goal from the planned one to where students are struggling, they will better understand the lesson, which can stimulate their future study. (Reflection Journal #7).

According to the statement, reflecting on my teaching practice has helped me gain a better understanding of my instructional decisions. Also, my reflections showed that my commitment to being responsive guided my teaching and reflections. Therefore, my identity as a responsive, reflective, and theoretically-grounded teacher is evidenced in my reflection journals.

While undertaking the practicum, I also took a research course on language classroom communication. The combination of the two courses represented the perfect comingling of theories and practice, and the theories I obtained in the research course enabled me to analyze my behaviors in teaching. On the one hand, compared to the team-teaching project, the practicum allowed for the comprehensive and systematic learning of the teaching activity. On the other hand, what was offered in the research course empowered me to understand and analyze teaching from a more theoretical lens. Building upon my previous learning in the program, I became more conscious and deliberate about using theories to guide my teaching practice. The combination of theories and practice developed my theoretically-grounded and reflective teacher identity, accelerating my dual identity as a researcher and a practitioner. When reflecting on my practicum experience, I presented my awareness of my growth as a language teacher:

[In the research course,] we have been focusing on the interactions between teachers and students and the strategies teachers use in [second/foreign language] L2 classrooms. [During the practicum,] I started to pay more attention to the interactions between [the instructor] and the students, the way she asks questions, and the types of questions she prompts. (Reflection Journal #3).

I also shared my opinions about the combination of theories and practice:

I feel good about connecting something I have learned from the textbook with authentic experiences. It might be easy to remember the theories, but it takes time to apply theories to practice effectively. (Reflection Journal #3).

As is revealed in the above statements, completing a practicum while taking the research course granted me the ability to bridge theories with practice; this promoted reasoned, informed teaching, which is essential to good teaching and better learning outcomes. Consequently, my identity as a theoretically-grounded teacher was strongly reinforced and improved through this experience.

In this master’s program, my teacher PI development was facilitated through collaboration with members of my CoP, including program faculties, course peers, and the ESL instructor I worked with. What I acquired through my learning, as well as comments and suggestions provided by the members of my CoP, strengthened my understanding of teaching and promoted my academic literacy development in language education.

#### 4.2.2. Rose’s continuous development in a doctoral program

My teacher PI development continued in the doctoral program I attended, which concentrates on language education. Learning from others in the community is vitally important for doctoral students, particularly during their early years in the program (Casanave, 2008). Therefore, the doctoral program served as another CoP whose members, including my academic advisor, course instructors, other faculty, my colleagues, and other doctoral students, supported my learning and development. Connecting and interacting with members of this CoP hastened my acculturation as a PhD student in the program, contributing to my growth as both a researcher and a language teacher. In the first semester, I took a course aimed at acculturating first-year doctoral students. Throughout the course, I kept weekly journals to reflect on my experiences during my first semester. In these journals, I made frequent notes of instances in which interacting with others (including students from various cohorts, TAs, course instructors, etc.) assisted me in figuring out solutions to challenges I encountered. For example, I remarked that “suggestions and assistance provided by others can play a crucial role for students, especially first-year doctoral students, in finding the right direction and performing properly within the academic community” (Final Paper for First-Year Doctoral Student Seminar). In this way, the value of CoP in fostering my learning and development was reinforced through my interactions with others.

During my doctoral studies, I developed my principal research interests in language teacher education and bilingualism. My academic literacy in both domains progressed by taking courses in theories and research methods, which, in turn, led to my PI development. Most courses I took required students to write responses to course readings. In these responses, students were expected to make connections between course readings and practice (e.g., teaching and research experiences). This practice of situating course readings in real-world contexts stimulated my critical thinking; likewise, transferring the readings into practice strengthened my comprehension of theories, promoted my thinking about how best to apply them to practice, and helped to develop my identity as both a researcher and a practitioner. Throughout the reflections and responses, I constantly emphasized the importance of developing the ability to implement and adjust theories in practice. I also noted the bidirectional character of this process by highlighting the crucial role that theories play in driving teaching practice:

After being trained [in graduate programs], I became a more effective teacher. My teaching became more logical. With all the rationales provided by theories, I could control my teaching in a better way, and I could also better understand, explain, and analyze my teaching under clearer theoretical guidance. (Pedagogical Autoethnography).

Although those theories make sense to me, they are still out there and not part of me. I need to internalize all the theories and make them a major part of my beliefs or philosophy. (Self-Reflection, January 26, 2016).

Throughout this process, I was mindful of adopting appropriate theories and approaches to steer my practice. My experience in the PhD program boosted my continual evolution into a more theoretically grounded language teacher.

The value of linking theory and practice was also emphasized in the teacher education courses I took as part of my studies. In my previous teaching experiences, I had engaged in reflective practice. However, learning relevant theories in the doctoral program underscored the significance of reflection. My belief in the value of reflective practice is consistent with my theoretical understanding. By examining relevant theories, I better understood my development as a language teacher from a more theoretical perspective. I also expanded my understanding of how reflection can positively assist teacher learning and development. In a pedagogical autoethnography assignment in a teaching and teacher education course, I reflected on my previous learning and teaching experiences. I described how many factors, including the members of my CoPs, shaped my path to becoming a language teacher. This activity continued my development as a reflective teacher by providing a space for reflection.

My belief that language teachers should be responsive and reflective was also reinforced by two courses I took in bilingualism and bilingual education. The theories and concepts I learned in both courses provided a solid foundation for my belief in the value of bilingualism in language teaching and advocacy for bilingual education—that “a better understanding of students’ cultural and linguistic backgrounds is crucial in customizing teaching strategies to meet the distinctive needs of students” (Statement of Teaching Philosophy). The theories and pedagogies I learned in these courses strengthened my belief in the instructional value of incorporating the native language (L1) into language learning in order to foster L2 development. For instance, in both courses, we discussed translanguaging. I had previously observed the use of students’ L1 in L2 courses and incorporated L1-related components in my teaching. Understanding translanguaging validated the integration of students’ L1 and relevant literacy skills in my prior teaching. Therefore, learning relevant theories allowed me to view my teaching activity from a theoretical perspective, supporting my teaching decisions and enhancing my instructional behaviors. In other words, the relevant theories I obtained in both courses extended my understanding and rationalized the benefits of translanguaging as a teaching pedagogy. In my reading responses in both courses, I constantly highlighted the critical roles that teachers play in accommodating students’ backgrounds, especially cultural and academic backgrounds, as teachers adapt their instruction:

It is important to realize the ample and useful resources that students’ L1 would function as, which leads to a positive ideology of students’ translanguaging. Meanwhile, [language] teachers also need to view students’ language study from a multilingual, rather than monolingual, perspective. Therefore, students’ language competence in their L1 has also to be considered during teaching. (Reading Response #7, Bilingualism & Biliteracy).

Teachers should have a good recognition of the knowledge that individual students bring to the classroom, meaning teachers need to dynamically and effectively assess students’ backgrounds in order to build up new learning. (Reading Response #9, Bilingualism & Biliteracy).

The above statements demonstrate the central place of responsiveness in my teaching philosophy.

While pursuing my doctoral degree, I worked as a writing consultant in a writing center. Distinct from the more conventional educational context of my PhD program, the writing center offered an alternative educational setting where I could apply theories to practice and develop my identity as a responsive and reflective language educator. In the writing center, I promoted and advocated for bilingualism and translanguaging by inviting clients sharing my L1 (Chinese) to decide which language they preferred to use in consultation sessions. For clients not sharing my L1, I offered freewriting and brainstorming activities in which their L1 always functioned as a helpful tool. These strategies allowed my clients to adopt different languages (L1 and L2) for communication and learning purposes, fostering more conversations and a closer rapport with my clients. Also, during brainstorming activities, I encouraged my clients to consider how their backgrounds impacted their development and education. In light of this, I provided more responsive feedback and assistance. Recalling my own development as a bilingual speaker and writer, I also stressed the importance of integrating L1 into L2 development, having realized the benefits and support that L1 provides. Consequently, my practice at the writing center was informed by my knowledge of theories of bilingualism, and my consultation decisions were also supported by pertinent theories. My commitment to being responsive and theoretically grounded in language teaching was also supported by clients’ comments. They appreciated the bilingual approaches I adopted, confirming the efficacy and relevance of the pedagogy I was employing and encouraging me to continue to develop the tools I was using in my approaches to language education. My practice and the theories I had learned were reconciled congruently.

My learning in the PhD program expanded my theoretical understanding of language education, thereby fostering my academic literacy development. They also validated my previous practice and reinforced my understanding of language teaching. Working in the writing center enabled me to connect theories with practice, allowed me to explore the appropriate theories through practice, and empowered me to adapt my practice accordingly. Hence, my development and work experience throughout my PhD studies imply a significant reciprocal relationship between the development of academic literacy and my teacher PI construction.

## 5. Discussion

In the previous section, we discussed the two participants’ academic literacy development experience, along with the formation of teacher PI in the master’s and doctoral programs in the United States. We have also delved into their professional narratives and synthesized the major themes from these descriptions.

### 5.1. Disciplinary literacy development and the construction of dual identity as both researcher and language teacher

Learning how to teach is a continuous process that requires ongoing improvement. One way to facilitate this professional growth is through continuous and meaningful teacher education, which has been proposed as a means to promote critical reflection in teaching institutions (Atay, 2008). The longitudinal records of both the novice teachers in this study illustrated their trajectories of academic literacy development and explained how these experiences had impacted their perceptions of their roles and identities in educational settings. Such perception, known as self-perception as teachers, refers to how teachers view or perceive themselves in their professional role as educators (Isbell, 2008). Both subjects found that the pedagogical knowledge and theories they acquired in various courses informed their teaching methods and developed their identities as researcher-teachers in the classroom. Informed by knowledge of bilingualism and translanguaging, Rose designed lessons and instructional activities in her teaching and consultation sessions and considered students’ backgrounds to provide responsive feedback and support. She also became reflective when she analyzed her teaching from a theoretical lens. According to Chapman (2009), this process of reflection can help teachers gain insights into the challenges they face in the classroom and explore alternative approaches to teaching that may be more effective. It seems that Rose balanced her roles as a teacher and researcher in her teaching practice, and her dual identity has been nourished and developed evenly throughout her experiences.

In Irene’s story, she employed knowledge from corpus linguistics, technology, and language courses, which guided her teaching as a student teacher in writing courses and in the development of corpus-based feedback systems for ESL learners. Students’ questions and feedback identified areas for improvement and research questions to pursue, thus helping her stay attuned to the reciprocal relationship between teaching and research. At the same time, Irene also admitted to sometimes finding it difficult to balance the demands of teaching and research in her classroom due to their competing expectations and resource and time management. She needed to adjust her research plans to prioritize the capabilities and needs of her students and fulfill her teaching responsibilities. Irene’s experience resonates with Tabach (2011), who believes that assuming the role of a researcher-teacher can simultaneously be a rewarding experience and a challenging task. Considering that the primary duty of a teacher is to ensure that the educational needs of their students are met, Tabach (2011) suggests that teachers should primarily assume the role of an instructor and minimize the influence of their role as a researcher. It is meaningful to further explore how novice teachers balance two distinct and demanding roles in the classroom to refine their teaching and research strategies while contributing to the broader understanding of educational theories and philosophies.

### 5.2. Sociocultural literacy development and micropolitics

One important aspect of a teacher’s PI concerns micropolitics, which refers to power relations, conflicts, and negotiations at the individual and small group levels within academic institutions. As Tan (2015) explains, micropolitics exists in organizations both overtly and covertly, as individuals and groups pursue informal and formal methods of gaining power. Zhu et al. (2018) found that strong micropolitical skills can help teachers build positive relationships with colleagues and mentors, advocate for their interests, achieve their goals, and contribute to a more productive and supportive academic community. In this study, Irene described her professional vulnerability and perceptions of micropolitics when she had a different view of language use in the class from her mentor during the teaching practicum. While she made attempts to justify her choice of using both Chinese and English as instructional languages to her mentor, she finally complied with her mentor and revised her teaching plan. Admittedly, there are hierarchical power dynamics embedded in teaching practicums, and novice teachers’ vulnerabilities may be heightened when encountering unbalanced power and competing interests (Bloomfield, 2010; Hallman, 2012). However, researchers have found that many teacher education programs fail to inform teachers of the micropolitical reality or help them develop micropolitical literacy, which allows teachers to know how to navigate power dynamics and relationships effectively and communicate with mentors and administrators in a constructive and collaborative manner (Kelchtermans and Ballet, 2002; Hong, 2010). To investigate micropolitics in the school environment, many studies (e.g., Hong, 2010) have focused on micropolitical relations between students and in-service teachers and between teachers and administrators; relatively few explore the micropolitics of novice teachers during teaching practicums (Zhu et al., 2018). From the lens of micropolitical theory, Zhu et al. (2018) revealed the unbalanced micropolitical realities of teaching practicums in China and their impact on Chinese novice teachers’ vulnerabilities. In comparison, our study uncovered a lack of training in sociocultural literacy in graduate programs in the United States and its influence on the participants’ perception of micropolitics and communication in relation to their teaching practice. The study adds to the limited research in this area by highlighting the lack of attention given to the micropolitical realities of teaching practicums in the United States and how this affects sociocultural literacy and communication among graduate student participants. The study also highlights the importance of providing novice teachers with better training and support in navigating power relationships that contribute to a more supportive, diverse, and productive environment.

### 5.3. Academic literacy development and changing teaching beliefs

In addition to changes in teacher PI, higher-order thinking, and discipline-specific social practices, novice teachers’ beliefs about teaching and being a teacher also changed. “Beliefs about teaching” refers to teachers’ personal views regarding the nature of learning, the role of the teacher, and the purpose of education (Hong, 2010). According to Fenstermacher (1994), teachers’ beliefs influence the instructions they give to students. When Rose entered her master’s program, she had observational experiences that teaching was teacher-centered. However, Rose’s views of successful language teaching were influenced by what she learned in her courses and during the teaching practicum. She recognized that student learning lay at the heart of the teaching activity, and the development of her belief in being responsive to student learning guided her teaching decisions. Rose continued to develop her teaching philosophy throughout her doctoral studies. Her conviction in the merits of reflective practice was shaped by the teacher education courses she took. These courses further strengthened her belief that language teachers should be responsive and reflective. Rose’s understanding of theories of bilingualism and translanguaging increased her belief and confidence in the instructional value of incorporating students’ L1 into language learning, and she also used this belief to guide her instructional decisions when advising writing center clients. Rose’s professional experience, in turn, promoted the growth of her teaching beliefs.

Rose’s story is in line with the argument that experiences in teacher education programs can overcome the influence of the “apprenticeship of observation” (Lortie, 1975) that teachers have experienced throughout their previous schooling experiences, reiterating the irreplaceability of teacher education programs in cultivating novice teachers and fostering their development (Johnson, 2016; Johnson and Golombek, 2016). In her story, Rose highlighted the influence she received from numerous reflection activities, which also contributed to the formation of her teaching beliefs and PI. Through reflection, teachers can thoroughly examine their teaching and externalize their thoughts. Their reflective narratives serve as an aid to self-awareness and illustrate their developmental trajectory (Golombek and Johnson, 2004). According to Rose’s story, experiences in graduate programs elevated her academic literacy and assisted in the formation of her teaching philosophy.

### 5.4. Academic literacy development and the changes of novice teachers’ self-efficacy and emotions

Self-efficacy and emotions are essential components of a teacher’s PI. Teachers’ self-efficacy refers to their judgments of their ability to plan and deliver effective instruction and support their students (Schepens et al., 2009). Emotions refer to teachers’ various subjective experiences in response to different situations at work, such as interactions with colleagues and students or instructional challenges (Hong, 2010). It is known that teachers with high levels of self-efficacy and good emotional management are more likely to use effective teaching practices and create a positive and inclusive learning environment (Hong, 2010). Many researchers have found that novice teachers often have a low sense of efficacy: they are not confident in managing the class and are particularly susceptible to stressful classroom situations (Ashton, 1985; Evans and Tribble, 1986). The longitudinal data in this study provide a more detailed depiction of how the self-efficacy and emotions of both participants changed in connection to their academic literacy development.

In terms of self-efficacy, Irene initially felt that she was not confident in managing the class and dealing with disruptions at the beginning of her teaching experience. This finding was consistent with the description of novice disciplinary teachers who are characterized by their overall lack of confidence in teaching performance and classroom management (Hong, 2010). However, in distinction from those who attributed their low self-esteem to the teacher training experience, Irene was worried about her English language competence and sociocultural knowledge in classroom instruction. Li (2007) described how NNES novice teachers often face challenges with their identity and cultural differences when they recognize themselves as both L2 learners and teachers instructing English as an L2. The confusion and challenges may lead to their low efficacy in the early years of teaching. However, Irene’s confidence in being a language teacher was boosted when she took more graduate courses and taught various ESL courses in the doctoral program. She explained the benefits to her teaching of being an L2 learner, as she could integrate the literacy skills she had learned into classroom instruction and show empathy to students. As suggested in the findings of this study, tracing novice teachers’ self-efficacy development in their early careers provides a more comprehensive understanding of it over time. This can help researchers identify patterns and trends in teacher self-efficacy development, as well as the factors that influence it. Teacher educators can also identify critical periods in the development of novice teachers’ self-efficacy and provide in-time support and interventions.

Regarding novice teachers’ emotions, Hong (2010) found that novice teachers did not experience as high a level of emotional burnout as in-service teachers. Meanwhile, they attributed stress and emotional burnout to individual teachers’ personal qualities and characteristics. In contrast to previous findings, Irene described a range of emotions she experienced in her master’s program, including excitement, anxiety, and stress. To manage her emotions, she needed to dress professionally, which set a positive tone for the classroom and helped her feel more in control of the classroom. When involved in more discipline-specific social practice as a teaching assistant in the doctoral program, she became better at coping with stress and challenges and maintained a positive outlook in the face of adversity. Rose, in her reflection journal, also described a moment that raised her anxiety and frustration during teaching. The reflection activity offered a positive opportunity, allowing her to critically analyze that moment and provoking her thoughts to shift the moment into a teachable one in the future. In this study, the longitudinal data recorded both participants’ nuanced emotional changes across their learning, teaching practicum, and student-teacher stages, revealing the means of dealing with frustration and stress in different disciplinary social practices. By understanding how novice teachers’ emotions evolve throughout their training and early teaching careers, researchers can gain insights into how best to support their emotional well-being and ultimately improve their teaching performance. Future work can further explore the relationship between L2 novice language teachers’ emotional regulation strategies and their teaching effectiveness.

## 6. Conclusion

This study uses the CoP (Wenger, 1998) as the theoretical framework and narrative inquiry as the methodology to explore the interrelationship between the PIs of two novice language teachers and their acquisition of academic literacy during their graduate studies in the United States. Both participants’ narratives were collected through course assignments, reflection journals, and other supplementary documents. The study found that active participation in CoPs facilitated the development of academic literacy and positively influenced the formation and growth of teachers’ PI. Specifically, the discipline-specific knowledge acquired during graduate programs contributed to the formation of researcher-teacher identities. The study also found that the development of academic literacy impacted the self-efficacy and emotions of language teachers. The narratives demonstrated the connection between academic literacy development and the evolution of teaching philosophy, but the study also revealed a lack of sociocultural literacy training at the graduate level. The findings suggest a relationship between the growth of academic literacy in professional CoPs and PI development. The study also highlights the critical role of teacher education programs in fostering novice teacher development. For future research, it is recommended to investigate the development of novice teachers’ dual identity as both researchers and teachers and to focus on how they balance the two distinct roles in classrooms. Additionally, it is suggested to further examine the emotional evolution of novice teachers throughout their training and early career stages to best support their emotional well-being and identity construction.

Given the nature of the narrative inquiry, the results of the current study cannot be extended to a larger context. However, through a thorough analysis of both novice teachers’ narratives, we comprehensively presented and described their PI developmental trajectories. Readers with similar backgrounds may find the conclusions relatable and applicable to their contexts with careful interpretations. For future studies, researchers may consider adopting a mixed-methods approach to incorporate quantitative data with qualitative analysis, particularly with regard to analyzing the extents of teacher PI development in various aspects.

## Data availability statement

The original contributions presented in the study are included in the article/supplementary material, further inquiries can be directed to the corresponding author.

## Ethics statement

The studies involving human participants were reviewed and approved by the University of Chinese Academy of Sciences. The patients/participants provided their written informed consent to participate in this study. Written informed consent was obtained from the individual(s) for the publication of any potentially identifiable images or data included in this article.

## Author contributions

Mo Chen: introduction, literature review, methods, data analysis, Irene’s story, discussion, revision. Wenli Zhang: abstract, theoretical framework, rationalization of adopting narrative inquiry, data analysis, Rose’s story, conclusion, revision. Qun Zheng: data analysis, revision. All authors revised the manuscript collaboratively. All authors approved the submitted version.

## Funding

The study was supported by the UCAS Foundation of Management Innovation in 2023 (Grant name: Investigating Modal Coherence in Timed Research Presentations. Grant number: E3E58946).

## Conflict of interest

The authors declare that the research was conducted in the absence of any commercial or financial relationships that could be construed as a potential conflict of interest.

## Publisher’s note

All claims expressed in this article are solely those of the authors and do not necessarily represent those of their affiliated organizations, or those of the publisher, the editors and the reviewers. Any product that may be evaluated in this article, or claim that may be made by its manufacturer, is not guaranteed or endorsed by the publisher.
